# Characterisation and genome sequence of the lytic *Acinetobacter baumannii* bacteriophage vB_AbaS_Loki

**DOI:** 10.1371/journal.pone.0172303

**Published:** 2017-02-16

**Authors:** Dann Turner, Matthew E. Wand, Yves Briers, Rob Lavigne, J. Mark Sutton, Darren M. Reynolds

**Affiliations:** 1 Centre for Research in Biosciences, Department of Applied Sciences, Faculty of Health and Applied Sciences, University of the West of England, Coldharbour Lane, Bristol, United Kingdom; 2 National Infections Service, Public Health England, Porton Down, Salisbury, Wiltshire, United Kingdom; 3 Laboratory of Applied Biotechnology, Department of Applied Biosciences, Ghent University, Ghent, Belgium; 4 Laboratory of Gene Technology, Biosystems Department, KU Leuven, Heverlee, Belgium; Centro Nacional de Biotecnologia, SPAIN

## Abstract

*Acinetobacter baumannii* has emerged as an important nosocomial pathogen in healthcare and community settings. While over 100 of *Acinetobacter* phages have been described in the literature, relatively few have been sequenced. This work describes the characterisation and genome annotation of a new lytic *Acinetobacter* siphovirus, vB_AbaS_Loki, isolated from activated sewage sludge. Sequencing revealed that Loki encapsulates a 41,308 bp genome, encoding 51 predicted open reading frames. Loki is most closely related to *Acinetobacter* phage IME_AB3 and more distantly related to *Burkholderia* phage KL1, *Paracoccus* phage vB_PmaS_IMEP1 and *Pseudomonas* phages vB_Pae_Kakheti25, vB_PaeS_SCH_Ab26 and PA73. Loki is characterised by a narrow host range, among the 40 *Acinetobacter* isolates tested, productive infection was only observed for the propagating host, *A*. *baumannii* ATCC 17978. Plaque formation was found to be dependent upon the presence of Ca^2+^ ions and adsorption to host cells was abolished upon incubation with a mutant of ATCC 17978 encoding a premature stop codon in *lpxA*. The complete genome sequence of vB_AbaS_Loki was deposited in the European Nucleotide Archive (ENA) under the accession number LN890663.

## 1 Introduction

Since 1966 more than 100 bacteriophages specific for the genus *Acinetobacter* have been reported in the literature, belonging to the families *Leviviridae*, *Myoviridae*, *Podoviridae* and *Siphoviridae* [1]. A total of 39 complete genome sequences are presently available for bacteriophages infecting *Acinetobacter* spp., representing less than 1% of all publicly available phage genome sequences. Two groups of *Acinetobacter* phages have recently been recognised as new genera by the International Committee for the Taxonomy of Viruses, the *Ap22virus* and *Fri1virus* belonging to the families *Myoviridae* and *Podoviridae*, respectively. While 19 siphoviruses infecting *A*. *baumannii* have been described, only four have been sequenced; YMC11/11/R3177 [2], Bphi-B1251 [3], IME_AB3 and the induced prophage vB_AbaS_TRS1 [4].

Interest in bacteriophages infecting species of *Acinetobacter* has increased in recent years, primarily due to the emergence of *A*. *baumannii* as a prominent multiple-drug resistant nosocomial pathogen, responsible for significant outbreaks of disease both in the UK and worldwide [5]. Due to a capacity to persist for extended periods in a dry environment [6] and resistance to treatment with disinfectants [7], combined with intrinsic and acquired antibiotic resistance [8], the control and therapeutic management of *A*. *baumannii* has become a pressing concern. A wide array of resistance mechanisms have been described for *A*. *baumannii* and strains with resistance to multiple antibiotics have been reported worldwide [9]. Colistin (polymixin B) has often been cited as the antibiotic of last resort for treatment of *Acinetobacter*, particularly in respiratory infections, but resistant strains have been widely reported in clinical settings [10]. Recent research suggests that *A*. *baumannii* appears to be able to readily adapt to colistin exposure [11], raising the probability of pan-drug resistance arising in this species in the near future.

The characterisation of bacteriophages facilitates a greater understanding of their biology, including host specificity, genomic diversity and adaptation to their bacterial hosts, facilitating their subsequent exploitation as therapeutic agents or as a resource for the development of genetic tools. In the present study, we report the isolation, characterisation and complete genome sequence of bacteriophage Loki (vB_AbaS_Loki), a new member of the *Siphoviridae* infecting *A*. *baumannii*.

## 2 Results

### 2.1 Virion morphology

**Fig 1:**
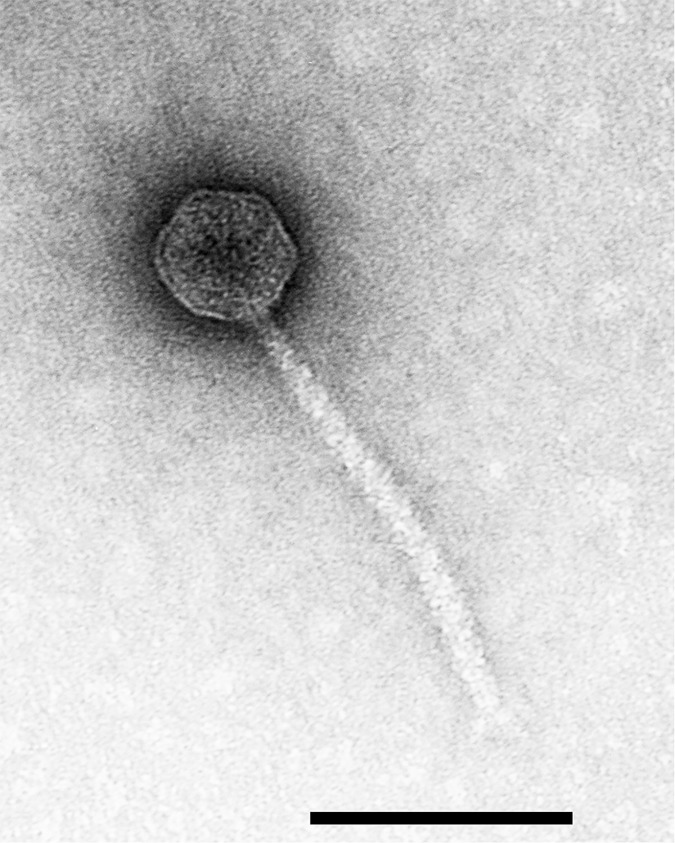
Transmission electron micrograph of Loki negatively stained with 2% w/v uranyl acetate. The scale bar represents 100 nm.

Loki was isolated from activated sludge following enrichment with *A*. *baumannii* ATCC 17978. Examination by transmission electron microscopy (Fig 1) revealed Loki to be a B1 siphovirus that resembles *Burkholderia* phage vB_BceS_KL1 [12]. Loki and KL1 share a similar morphology to the flagellum-specific Enterobacteria phage chi (χ) but lack the characteristic single long terminal tail fibre of this phage (H.-W. Ackermann, personal communication). The capsid is isometric, measuring 57 ± 4 nm across opposite apices. The non-contractile tail exhibits transverse striations, measures 176 ± 3 nm in length and 10 ± 0.9 nm in diameter with short tail spikes present at the tail terminus.

### 2.2 Adsorption and one-step growth

**Fig 2:**
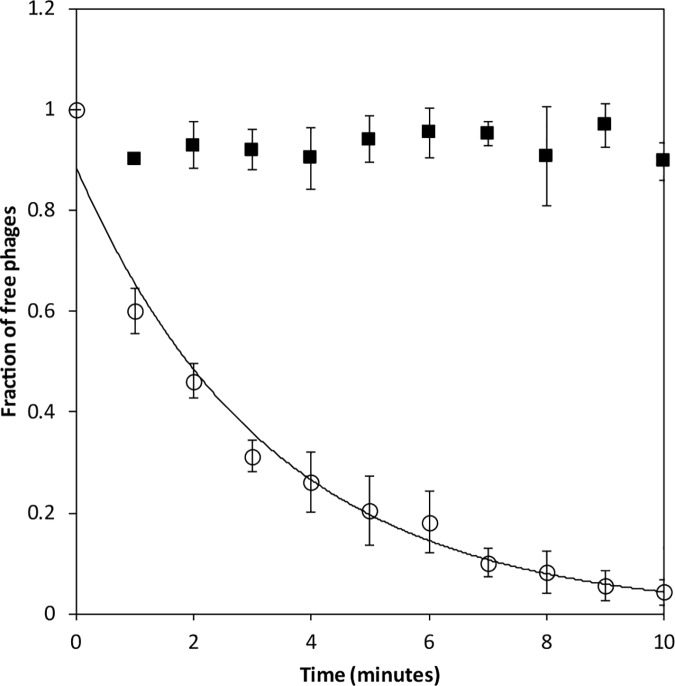
Adsorption of Loki to *A*. *baumannii* ATCC 17978 (open circles) and lpxA mutant (filled squares). The fraction of free phages remaining in solution is plotted over time. Error bars denote standard deviation (n = 5).

Under conditions of one-step growth, the latent period was determined as 40 minutes with a rise period lasting a further 30 minutes yielding a burst size of 43 p.f.u per infective centre. An average burst size of 40 ± 5.8 p.f.u. per infective centre was determined for single burst experiments. The eclipse period was not determined. Loki exhibited an adsorption rate constant of 1.32 x 10^−8^ ml/min to cells of *A*. *baumannii* ATCC 17978 at 30°C (Fig 2). Adsorption was abolished using a mutant of ATCC 17978 that had undergone a single point mutation in *lpxA* (E216Stop] following exposure to sub-MIC concentrations of colistin [11]. The introduction of a premature termination codon results in the truncation of LpxA from 262 to 216 amino acids. LpxA is a critical enzyme in the biosynthesis of lipid A and mutations to this gene have been demonstrated to result in the complete loss of lipopolysaccharide (LPS) biosynthesis in *A*. *baumannii* ATCC 19606 and a concomitant increase in resistance to colistin [13]. This evidence suggests that the host cell surface receptor utilised by Loki might be a component of LPS. ATCC 17978 possess a smooth form of LPS in addition to an exopolysaccharide capsule [14]. Phages of Gram-negative bacteria use a variety of cell surface structures that include flagella, pili, outer membrane proteins as well as the O-antigen, inner and outer core polysaccharides of LPS for host recognition [15,16].

### 2.3 Host range and efficiency of plating (EOP)

Loki forms small, turbid plaques on *A*. *baumannii* ATCC 17978 of average size 0.5 ± 0.1 mm after incubation at 30°C. Plaques ceased to expand after 16 hours of incubation. Titres determined using overlay agar plaque assays were unaffected after treatment of phages with chloroform. The requirement for calcium ions to expedite plaque formation was investigated by varying the concentration of calcium chloride added to overlay and underlay agar. The ability of Loki to form plaques was strictly dependent upon the presence of Ca^2+^, whereas titres were unaffected by the presence or absence of Mg^2+^ at 10 mmol l^-1^. Overlay plates prepared without 5 mmol l^-1^ CaCl_2_ yielded no plaques and omission of CaCl_2_ from the bottom agar reduced the number of plaques by approximately 50% (Table 1).

**Table 1:** Ca^2+^-dependent infection of *A*. *baumannii* ATCC 17978. Titres are the mean of triplicate assays. Values in parenthesis are the efficiencies of plating compared with the titre on *A*. *baumannii* ATCC 17978 in the presence of 5 mmol l^-1^ Ca^2+^.

p.f.u ml^-1^ *A*. *baumannii* ATCC 17978		
+5 mmol l^-1^ Ca^2+^ (overlay and underlay agar)	+5 mmol l^-1^ Ca^2+^ (overlay agar alone)	0 mmol l^-1^ Ca^2+^
7.43 x 10^8^ (1)	3.64 x 10^8^ (0.49)	No plaques (0)

A number of *Siphoviridae* infecting both Gram-positive and Gram-negative hosts have been demonstrated to depend upon the presence of Ca^2+^ ions, including *Mycobacterium* phage L5 [17], *Escherichia* phage T5 [18], *Bacillus* phage SF6 [19] and the 936 group of *Lactococcus* phages [20]. The requirement for Ca^2+^ has been shown to affect either adsorption, transfer of the phage genome or the formation of progeny virions in these phages. Notably, while zones of clearing were evident on spot plates of the propagating host, individual plaques were not observed on overlay plaque assays at an incubation temperature of 37°C. A similar lysis phenotype was previously observed by Lynch et al., during their characterisation of the related *Burkholderia cepecia* phage KL1 [12].

Loki exhibited broad tropism under spot plate conditions at 30°C, forming zones of clearing against type strains and clinical isolates of *A*. *baumannii* encompassing all three international clonal lineages (S1 Table). Zones of clearing were observed for 36 of 38 strains (95%) of *A*. *baumannii* in addition to a single strain of *A*. *lwoffii* and *A*. *baylyi*. This broad host range was not reflected in subsequent titrations to determine EOP where plaque formation was not observed at any dilution for any of the *Acinetobacter* strains tested other than the propagating host. The discrepancy between these results confirms the necessity of EOP when assessing the host range of a new phage isolate. Efficiency of plating represents a more stringent assessment of host range, since with low number of phages plaques can only be formed by productive infection, that is, through the production and release of viable progeny virions through cell lysis. The zones of clearing observed under spot plate conditions may have arisen as the result of adsorption of large numbers of phage causing destabilisation of the outer membrane; the classical definition of lysis from without [21]. Alternatively, these zones of clearing may have arisen from the presence of bacteriocins in the phage preparation [22] or potentially, abortive infection resulting in the inhibition of phage replicative processes [23]. Loki did not cause lysis against either *Escherichia coli* K12, *Pseudomonas fluorescens* NCIMB 9046, *Burkholderia cepecia* NCIMB 9088 or *Klebsiella pneumoniae* NCTC 10896.

### 2.4 Genome sequence

Sequencing of phage Loki yielded a single contig with a high average coverage of 3,435x. Loki has a linear dsDNA genome of 41,308 bp with a G+C content of 44.35%, slightly higher than that exhibited by most *A*. *baumannii* genomes, for which the GC contents of available genome sequences range between 38.7% and 42.6%.

Undigested Loki genomic DNA yielded a single high molecular weight band estimated at 42,100 bp when resolved by PFGE, approximately 800 bp greater than the sequenced genome assembly. The sequence assembly was circular, indicating that the Loki genome is either circularly permuted or has a non-permuted terminal redundancy, e.g. direct terminal repeats. Several types of termini among the *Caudovirales* have been studied including, but not limited to, single stranded 3’ and 5’ cohesive ends, short and long non-permuted direct terminal repeats, covalently bound terminal proteins, terminal host sequences and circularly permuted direct terminal repeats [24,25].

Banding patterns after digestion of Loki genomic DNA with restriction enzymes were in agreement with those predicted *in silico* from the genome sequence, assuming a circular conformation (Figure A in S1 File). In order to discount the presence of cohesive ends at the genome termini, Loki genomic DNA digested with either BmtI, BsrGI or BclI was denatured by heating at 80°C followed by rapid or slow cooling (Figure B in S1 File). If the genome has cohesive ends, the two restriction fragments possessing the cohesive termini will anneal in the slow cooled sample and form a single larger fragment [24]. No alteration to the restriction profile was observed between the rapid and slow cooled samples. Additionally, treatment of Loki genomic DNA with T4 DNA ligase prior to digestion did not alter the pattern of restriction fragments (Figure C in S1 File). If cohesive termini were present, ligation would cause the two restriction fragments containing the termini to appear as a single fragment the sum of the respective sizes. Cohesive ends can therefore be excluded. Time limited digestion with the exonuclease BAL-31 followed by digestion with BmtI, BsrGI, BclI or SspI resulted in an even, simultaneous degradation of all restriction fragments (Figures D-G in S1 File). These results discount the presence of fixed termini, such as direct terminal repeats, where a progressive shortening of two restriction fragments containing the fixed termini due to exonuclease activity would have been observed [26]. The simultaneous degradation of all restriction fragments indicates that the position Loki genomic termini are variable, representing a population comprised of many different end positions. Circularly permuted genomes are characteristic of a head-full packaging strategy, where the packaged DNA length is between 102 and 110% of the total genome length, resulting in terminal redundancy [24]. No submolar packaging (*pac*) fragment associated with the terminase initiation cleavage site was observed on electrophoresis gels. Taken together, these data suggest that the Loki genome is circularly permuted and terminally redundant. Head-full packaging has been associated with generalised transduction, a characteristic that has the potential to facilitate horizontal transfer of host DNA [24].

The genome sequence is opened at the small terminase subunit, according to convention [24]. A total of 51 open reading frames (ORFs) were predicted, representing a coding percentage of 95.4% at a density of 1.23 genes per kb. No tRNAs or rRNAs were identified and putative functions could be assigned for 31 (60%) of the ORFs after analysis using BLASTP, HHblits, HHsearch, Pfam and InterProScan (S2 File). No putative gene products with similarities to proteins involved in lysogeny or host conversion were found, which appears to confirm that Loki is a strictly lytic phage.

**Fig 3:**
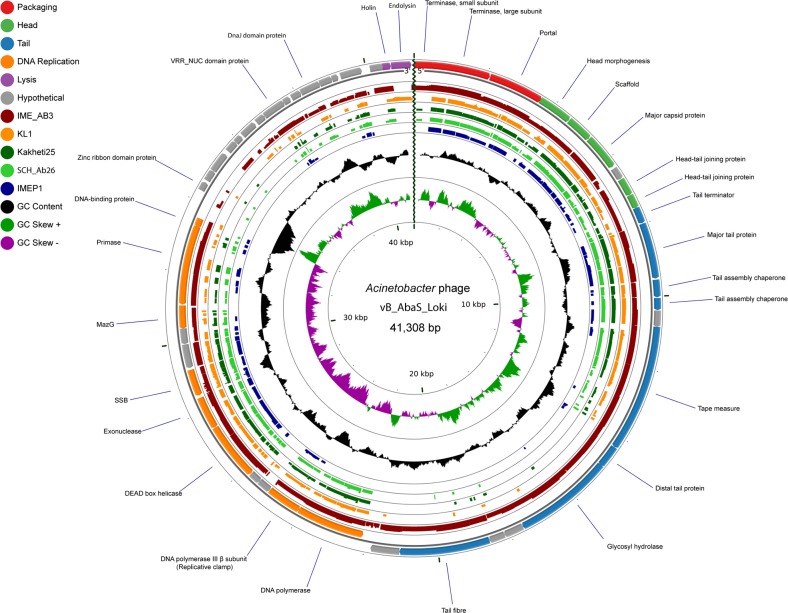
Comparative genome map of Loki prepared using the CGView Comparison Tool [29]. The outer ring illustrates ORFs encoded by Loki, coloured according to putative function as shown in the key. TBLASTX was used to compare sequence similarity with the related phages IME_AB3 [KF811200], KL1 [JF939047], vB_Pae_Kakheti25 [JQ307387], SCH_Ab26 [HG962376] and IMEP1 [KP162168], shown in red, orange, green, light green and blue, respectively. GC content is depicted in black, while positive and negative GC skew is shown as green and purple, respectively.

As has been observed for many *Siphoviridae* [27,28], the Loki genome is organised into discrete functional modules (Fig 3) containing genes involved in virion morphogenesis (gp01-gp21), DNA replication (gp22-gp33) and a final module encoding early genes and those involved in cell lysis (gp34-gp51).

#### 2.4.1 Packaging, morphogenesis and structural proteins

**Fig 4:**
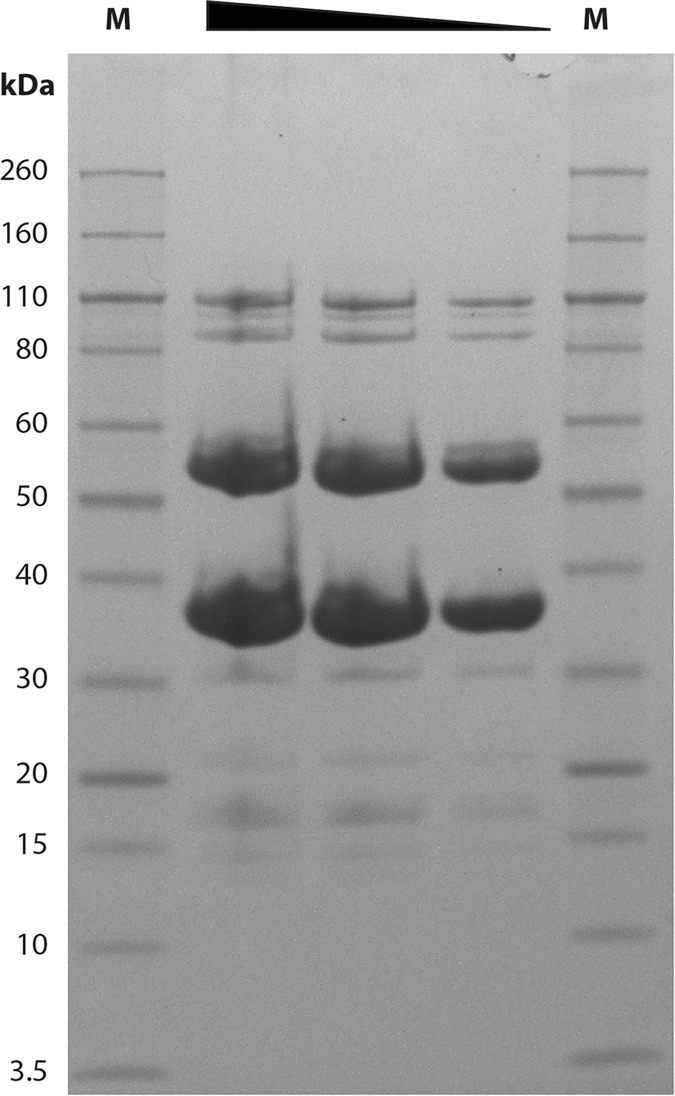
1D SDS-PAGE of structural proteins from CsCl-purified Loki virions. Lanes containing protein standard ladders are labelled M and the sizes of markers in kilodaltons (kDa) are shown on the left. The triangle depicts decreasing concentrations of loaded protein.

Genes encoding assembly chaperones and the mature virion structural protein components are located in a continuous module comprising 21 ORFs, representing 39.2% of the total encoded genes. The genes in this module follows the classical *Siphoviridae* organisation; genes encoding the DNA packaging apparatus are followed by those encoding the head, head-tail joining, the tail and adsorption apparatus [27]. Analysis of CsCl-purified virions using 1D SDS-PAGE resolved 11 protein bands, representing virion structural components, with molecular weights ranging from 12 to 108 kDa (Fig 4). The two predominant bands at 54 and 35 kDa are suggestive of major capsid and major tail proteins, respectively and correspond to the predicted molecular weight of these proteins.

Based upon searches conducted using BLASTP and HHsuite (S2 File) the functions of 16 gene products (76%) involved in virion structure and morphogenesis could be identified. The DNA packaging apparatus of tailed phages consists of a heterodimeric terminase composed of small and large subunits where the small subunit is responsible for DNA binding and the large terminase mediates prohead binding and cleavage of concatameric phage DNA into individual genome units. The Loki small terminase subunit was assigned on the basis of synteny and homologs identified using PSI-BLAST. The large terminase contains a Pfam Terminase_6 domain (PF03237) and is encoded immediately upstream of the portal vertex protein (DUF4055). Gene products identified to play a role in capsid assembly include a SPP1 gp7 family morphogenesis protein (gp04) (PFAM PF04233), scaffold (gp05) and major capsid protein (gp06). Two proteins were identified to play a putative role in head-tail joining, gp08 and gp09 that showed similarity to the connector proteins gp6 of HK97 (PDB 3jvo) and gpFII (PDB 1k0h) of Lambda, respectively [30,31]. Gp10 shows significant similarity to the tail terminator proteins gp17 (PDB 2lfp) of *Bacillus* phage SPP1 and the minor tail protein gpU (PDB 3fz2) of Lambda. These proteins play an essential role in tail assembly, terminating tail polymerisation and providing a surface for interaction between the mature phage head and tail [32,33].

The major tail protein (gp11) was assigned on the basis of synteny and a weak match to the major tail protein, GpV, of Lambda (PDB 2k4q). In many tailed phages, correct formation of the tail is dependent upon the expression of two chaperones, one of which is produced as a fusion product by appending an additional C-terminal domain via a programmed translational -1 frameshift. Tail chaperones produced through frameshifts have been confirmed experimentally for phages λ, P2, L5, TM4, phiC31, HK022, and HK97 [34–37]. The presence of a frameshift motif AAAAAAC immediately adjacent to the stop codon of gp12 would suggest a similar frameshift event may occur in Loki resulting in a fusion product of gp12 and gp13. An identical motif is found in the tail assembly chaperone of IME_AB3 (locus_tag: IME_AB3_31).

A further gene product predicted to be involved in tail assembly protein, gp14, is encoded between the tape measure and assembly chaperones for which HHsearch identified a HK97 gp10-like domain (PF04883) with high probability. The putative tape measure, gp15, was identified by the presence of a conserved domain (IPR013491 tape_meas_nterm), a predicted coiled coil structure and by homologs in other phages and prophages identified by BLASTP. Gene products encoded by gp16 through gp21 are predicted to encode products that comprise the distal tail tip complex and facilitate adsorption to host cells. PSI-BLAST searches linked gp16 with the distal tail protein pb9 of phage T5 and other T5-like phages EPS7, SPC35 and Akfv33 after three iterations. In addition, HHsearch returned a significant match to pdb9 (PDB: 4jmq). The T5 pdb9 protein has been suggested to represent a large family of distal tail proteins that are found in numerous siphoviruses and which form a hexameric ring that serves to connect the tail tube to the adsorption apparatus [38]. The function of gp17 is not clear but HHsearch returned matches to hydrolases in the pdb70 and pfam27 HHsuite databases. Gene product 18 is also likely to form part of the tail tip complex, indicated by a weak match to the gp44 baseplate hub protein of the temperate myovirus Mu (PDB: 1wru) and a significant match to the lambda minor tail protein L domain (PF05100) using HHsearch. For gp19, searches conducted with HHsearch against the Pfam28 and pdb70 databases returned matches to bacteriophage peptidoglycan hydrolases and bacterial cell wall hydrolases, respectively, that might suggest a role for gp19 in facilitating translocation of the virion DNA across the bacterial cell wall. The putative tail fibre, gp20 was assigned this function due to the presence of a fibronectin-like domain (Pfam: PF13550 Phage-tail_3) commonly associated with phage tail fibres. No function could be assigned to gp21.

#### 2.4.2 DNA replication gene module

Putative functions could be predicted for nine gene products encoded by the replication cluster. Loki encodes a putative family B DNA polymerase (Gp22), DNA polymerase III β subunit (gp23), helicase (gp26), single stranded DNA binding protein (gp28) and primase (gp32). In addition Loki encodes a putative exonuclease (gp27), a DUF3987 domain protein (gp32) and a protein linked to a SprT-like and peptidase domains by HHsearch (gp24). A notable feature is the presence of a MazG domain protein (gp31) within the DNA replication module. In *E*. *coli* MazG is a nucletotide pyrophosphohydrolyase that is able to hydrolyse all canonical nucleoside triphosphates, has been demonstrated to act as a regulator of the stringent response by interaction with Era and inactivation of ppGpp [39]. Proteins containing the MazG domain have previously been identified in marine phages as well as those infecting diverse bacterial genera that include phages closely related to Loki [12,40–43]. While no functional assays have yet been performed to demonstrate that these phage proteins act as MazG homologs, it has been suggested that these MazG domain proteins might act to extend the logarithmic phase of bacterial growth, facilitating the production of progeny virions through the reactivation of metabolic pathways that are usually suppressed under conditions of nutrient starvation [41].

#### 2.4.3 Early/Lysis gene module

Putative functions could be assigned for only five from a total of 18 ORFs present in the early/lysis gene module. A zinc ribbon domain (PF13248) protein, gp35, was identified using HHsearch and gp42 encodes a VRR_NUC domain. An incomplete C-terminal cysteine-rich DnaJ domain (IPR001305) was predicted for gp45 by InterProScan, with two conserved CXXCXGXG motifs while HHsearch identified a significant match to the antitermination protein Q of lambda (PDB: 4mo1).

Genes identified to play a role in host cell lysis consisted of a putative class I holin (gp50) with two predicted transmembrane domains encoded immediately upstream of the putative endolysin (gp51) that contains an N-acetylmuraminidase (pfam: PF09374) and a C-terminal peptidoglycan binding domain (Pfam: PF05838). Modular endolysins are relatively rare in phages infecting Gram-negative hosts, most produce endolysins comprised of a single domain that acts upon a specific peptidoglycan bond. All modular endolysins active against Gram-negative bacteria described to date contain a conserved repeat sequence in the peptidoglycan-binding domain [44]. The Loki endolysin contains a single copy, albeit less conserved, of this motif.

### 2.5 Relationships between Loki and other *Siphoviridae*

**Fig 5:**
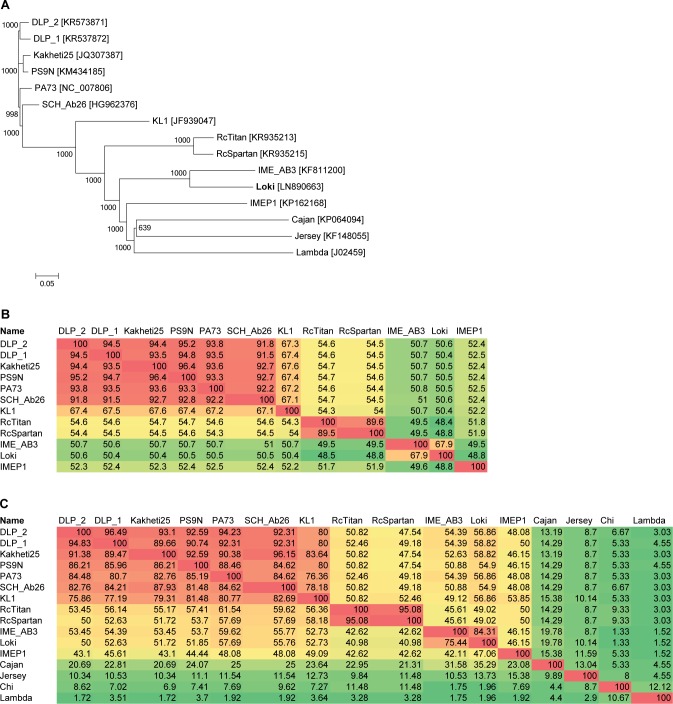
(a) Phylogenetic tree of co-linearised whole genome nucleotide sequences of Loki and phages linked by TBLASTX searches. The neighbour joining tree was constructed using ClustalX with 1,000 bootstrap replicates. The scale bar represents substitutions per site. (b) Percent nucleotide identity matrix determined using EMBOSS Stretcher. (c) Matrix showing the percentage of shared genes determined using CoreGenes 3.5. Cells are coloured from green to red to show increasing similarity. Phages Jersey, Lambda, Cajan and Chi were included as outliers.

Searches conducted using dc-megablast and megablast revealed significant nucleotide similarity between Loki and *Acinetobacter* phage IME_AB3 and these two phages exhibit significant synteny across the entire genome. Additional relationships with *Burkholderia* phage vB_BceS_KL1 [12], *Paracoccus* phage vB_PmaS_IMEP1, *Stenotrophomonas* phages DLP1 and DLP2 [43], *Rhodobacter* phages RcTitan and RcSpartan [45], as well as *Pseudomonas* phages vB_Pae_Kakheti25 [46], vB_PaeS_SCH_Ab26 and PA73 [47] were also identified using TBLASTX. To perform pairwise comparisons, genomes were co-linearised to start at the small terminase subunit and aligned using ClustalX (Fig 5A) and EMBOSS Stretcher (Fig 5B). At the protein level, CoreGenes3.5 was used to calculate the percentage value of gene products conserved between each phage (Fig 5C).

Each of the phages linked by TBLASTX possess a similar genome size (41.3 to 43.1 kb) and number of ORFs (51 to 58). Each phage exhibits conservation of structural genes encoding the DNA packaging apparatus, virion head and tail but possess distinct genes encoding the tail tip complex and adsorption apparatus reflecting their different host tropism. Key replicative gene products including a DNA polymerase, helicase, primase, single stranded DNA binding protein and MazG are also conserved between the phages. The gene products encoded within the early/lysis gene module are significantly more divergent, with only the putative holin conserved in each phage. The EMBOSS stretcher and CoreGenes data suggest that KL1, SCH_Ab2, Kakheti25, PS9N, PA73, DLP_1 and DLP_2 represent a discrete clade of *Siphoviridae* united by a minimum 67.1% sequence identity and 75.9% shared orthologous proteins. This cluster of phages are more distantly related to Loki and IME_AB3; the two groups are linked by a minimum of 50% nucleotide sequence identity and 49% protein homologs.

## 3 Discussion

The reduction in cost of genome sequencing has led to a marked increase in the number of complete phage genomes deposited in the international sequence databases (EMBL, GenBank and DDBJ). This wealth of sequence data has demonstrated the enormous diversity of the tailed phages and led to the proposal of new taxonomic relationships between phages isolated at disparate times and geographic locations. Significant work has recently been reported defining taxonomic relationships within the *Siphoviridae* and for bacteriophages infecting the Enterobacteriaceae [48–50]. In contrast to phages infecting other Gram-negative bacteria, particularly those infecting genera of the Enterobacteriaceae, few *Acinetobacter* phages have been sequenced.

Loki and IME_AB3 are two closely related representatives of a novel group of siphoviruses known to infect *A*. *baumannii* that exhibit little nucleotide similarity to other *Siphoviridae* in the extant sequence databases. However, the analysis of gene products demonstrates that these two *Acinetobacter* phages share a significant number of genes involved in virion morphogenesis and DNA replication with a small group of phages infecting *Pseudomonas*, *Paracoccus*, *Burkholderia* and *Rhodobacter* species. Loki and IME_AB3 are distinguished primarily by the presence of different small hypothetical proteins situated in the early/lysis gene module. Due to the lack of functional inferences that could be obtained using bioinformatics approaches and from the genomic location we posit that these proteins might be expressed early in infection and be involved in the hijack of host cell functions. A second distinguishing feature is the structure of the endolysin. In Loki the endolysin is modular, comprising a conserved peptidoglycan-binding domain and an N-acetylmuraminidase domain. In contrast the endolysin encoded by IME_AB3 is predicted to encode a single glycoside hydrolase family 19 domain.

A common feature of bacteriophage genomes is the presence of small genes of unknown function, many of which exhibit little or no similarity to entries in the extant sequence database [51]. The establishment of a productive lytic infection depends upon the interaction between phage- and host-encoded proteins in order to inhibit, regulate and/or subvert a variety of cellular processes to create an optimal environment for the production of progeny virions [52]. Recent work on bacteriophages infecting *Pseudomonas aeruginosa* has leveraged RNA sequencing, protein-protein interaction and metabolomics studies to identify proteins expressed early in infection involved in host cell takeover [53–56]. These studies have revealed a number of inhibitory phage proteins that target an array of host cellular processes including transcription, RNA degradation, DNA replication, cell division and fatty acid and riboflavin biosynthesis pathways. Such genome mining approaches are important not only for the elucidation of bacteriophage biology but also to leverage rational drug design by mimicking the mechanism of action of antibacterial phage proteins in an era where the rate of discovery of new antibiotics has slowed and antibiotic resistance is increasing. Given the prevalence and breadth of antimicrobial resistance in *A*. *baumannii*, similar approaches are required to elucidate the biological mechanisms underlying productive phage infection.

## 4 Materials and methods

### 4.1 Bacteriophage isolation and purification

In June 2015 a fresh sample of approximately 500 ml of activated sludge from Cam Valley sewage treatment works was provided by Wessex Water Services Ltd. Bacteriophage Loki was isolated by incubation of 5 ml of activated sludge diluted in 5 ml of double-strength LB broth (Invitrogen, UK) supplemented with 10 mmol l^-1^ MgSO_4_ and 5 mmol l^-1^ CaCl_2_. A 200 μl volume of an overnight batch culture of *A*. *baumannii* ATCC 17978 was added and the enrichment sample was incubated at 30°C for 18 hours with shaking at 150 rpm. Following incubation, the enrichment sample was centrifuged (8,000 x *g*, 10 minutes) to pellet bacterial cells, filtered (0.45 μm pore size) and assessed for the presence of bacteriophage by overlay plaque assay [57]. Clonal preparations were made by stab sampling an individual plaque followed by elution in SM buffer and plating on overlay agar.

High titre phage stocks were prepared by broth lysis. In brief, host bacteria were grown in LB broth at 30°C with orbital shaking at 150 rpm to an OD_600nm_ of 0.1 (c. 5 x 10^7^ c.f.u. ml^-1^). Bacteriophages were added to yield a multiplicity of infection of 0.1 and subsequent growth and lysis of cultures was monitored by measurements of optical density at 30 minute intervals. Residual bacteria were killed by the addition of chloroform (final concentration of 1% v/v) and the crude lysates were treated with DNase I and RNase A (Sigma Aldrich, UK) at final concentrations of 1 μg ml^-1^ for 1 hour at 37°C prior to the removal of bacterial debris by centrifugation at 11,000 x *g* for 10 min at 4°C. The supernatant was filtered (0.2 μm pore size) and bacteriophages precipitated by addition of PEG 8000 (10% W/V) and 1 mol l^-1^ NaCl [58]. Precipitated phages were recovered by centrifugation at 11,000 x *g* for 20 minutes and residual PEG was removed by centrifugation with an equal volume of chloroform at 3,000 x *g* for 10 minutes. Pure preparations of bacteriophages were obtained by isopycnic centrifugation using a SW40Ti rotor (Beckman Coulter, UK) at 160,000 x *g* for 24 hours at 4°C in 0.75 g ml^-1^ cesium chloride. Following centrifugation, bands (approximately 1 ml) were recovered using a syringe and dialysed using a 10 kDa dialysis cassette (Thermo Fisher Scientific, UK) against two 500-fold volume changes of SM buffer to remove residual CsCl and stored at 4°C in SM buffer (50 mmol^-1^ Tris, 100 mmol l^-1^ NaCl, 8 mmol l^-1^ MgSO_4_, pH7.5).

### 4.2 Electron microscopy

Transmission electron microscopy was performed as described previously [59]. CsCl-purified bacteriophages were pelleted by centrifugation at 25,000 x *g* for 1 hour and washed in 0.1 M ammonium acetate (pH 7.2; Sigma-Aldrich, UK). Samples were deposited on 200 mesh formvar carbon-coated grids (Agar Scientific, UK) and stained with either 1% (w/v) uranyl acetate solution (pH 4.5; Sigma-Aldrich, UK) or 2% (w/v) sodium phosphotungstate (pH 7.0; Sigma-Aldrich, UK). All specimens were examined using a Philips CM10 transmission electron microscope operated at 60 kV. Magnification was calibrated using T4 tails and virion dimensions established by measurement of 20 intact particles.

### 4.3 Adsorption and one-step growth

Phage adsorption to host cells was performed as described previously [57]. Host strains were grown in LB to an OD_600nm_ of 0.1 (approx. 5×10^7^ c.f.u. ml^−1)^, serially diluted and enumerated using a spiral plating system (Don Whitely Scientific, UK). Bacteriophages were added to cultures to yield a final concentration of 5×10^4^ p.f.u. ml^−1^ (t = 0). At 1 min intervals, 50 μl was transferred to 950 μl SM buffer saturated with chloroform and stored on ice. Samples were titrated for unabsorbed bacteriophages by triplicate overlay plaque assays. Absorption rate constants (k) were calculated as −m/N, where m represents the slope of linear regression of the natural logarithm of the free phage titre over time and N the initial bacterial density in c.f.u. ml^−1^.

For one-step growth experiments, bacteriophages were added to host bacteria at a multiplicity of infection of 0.05 and allowed to adsorb for 5 minutes, then centrifuged at 13,000 x *g* for 1 min. The supernatant containing unabsorbed bacteriophages was discarded and the pellet suspended in 10 ml of fresh LB broth, diluted to 10^−2^ and incubated at 30°C. Samples were taken at 5 minute intervals over a 2-hour period and titrated by triplicate overlay plaque assays. Infected cultures for single burst experiments were prepared in the same way, diluted to 10^−7^ and 0.5 ml aliquots distributed to 50 tubes. After incubation for 2 hours at 30°C plaque overlay plates were prepared using the entire volume from each tube. The expected number of infected cells was calculated using the Poisson distribution from the observed number of plates yielding no plaques. For the three independent repeats the expected number of cells were 11, 5 and 6, equivalent to 0.22, 0.11 and 0.13 infected cells per 0.5 ml aliquot.

### 4.4 Determination of host range

Bacteriophage host range and efficiency of plating (EOP) were determined by the standard double agar layer plating technique [60]. Prior to testing, bacteriophages were adjusted by dilution to yield a titre of 10^10^ p.f.u. ml^−1^ on their respective propagating host. Spot tests were performed using square 120 mm plates (Greiner BioOne, UK) containing LB agar were subdivided to yield a 6×6 grid, to which 8 ml LB overlay agar containing 200 μl of an exponential phase culture was overlaid. To each section of the grid, 5 μl aliquots of bacteriophage from a tenfold dilution series were spotted onto the surface of the overlay agar so that each plate assessed a range of phage concentrations (10^10^ to 10^3^ p.f.u. ml^-1^). Phage samples were allowed to absorb into the overlay agar prior to incubation at 30°C and examined for plaque formation after 24 hours. For EOP testing, overlay plaque assay plates were prepared containing 10 μl of serial dilutions of bacteriophage and 150 μl of exponential phase bacterial cultures. Following overnight incubation at 30°C, the relative EOP was expressed as the ratio between the titre in p.f.u. ml^−1^ for a given isolate and the titre for the propagating host from overlay plaque assays.

### 4.5 DNA electrophoresis

Genome size was estimated by pulsed-field gel electrophoresis. Agarose plugs (1% w/v agarose) were prepared containing bacteriophages at an approximate concentration of 1 x 10^8^ pfu ml^-1^ and lysed by addition of proteinase K (New England Biolabs, UK) and SDS to final concentrations of 50 μg ml^-1^ and 0.5% w/v, respectively, in 10 mM Tris, 50 mM EDTA and incubating for 2 hours at 54°C. Agarose plugs were washed three times by soaking in Tris-EDTA buffer (10 mM Tris, 1 mM EDTA; Sigma Aldrich, UK) for 1 hour. Gels (1% w/v agarose in 0.5x Tris Borate EDTA (TBE) buffer) were run at 6 V cm^-1^ for 14 hours in 0.5x TBE buffer at 15°C with pulses of 1 to 12 seconds using a CHEF DRII electrophoresis unit (BioRad, France). DNA size standards were provided by a low-range PFGE ladder (New England Biolabs, UK).

Restriction digests of phage genomic DNA were separated using either 0.8% w/v agarose gels run at 4.5 V cm^-1^ or 0.6% w/v agarose gels at 3.5 V cm^-1^ in 1x TAE buffer (pH 8.0) as appropriate. DNA size standards were provided using 2-log and 1 kb extend DNA ladders (New England Biolabs, UK). Bands were visualised by staining with SYBR Safe (Life Sciences, UK) and gels imaged using a FluorChem Q (ProteinSimple, UK) and analysed using ImageJ [61].

### 4.6 1D SDS-PAGE

Virion structural proteins were concentrated using methanol-chloroform extraction [62] from CsCl-purified samples. Extracted proteins were suspended in lithium dodecyl sulphate sample buffer (Invitrogen) prior to heating at 70°C for 10 minutes. Protein separation was performed using a NuPAGE mini-gel system and Novex 4–12% Bis-Tris gels in MES–SDS running buffer at 200 V alongside Novex Sharp unstained protein standards (Life Technologies, UK). SDS-PAGE gels were stained using SimplyBlue Safestain (Life Technologies, UK).

### 4.7 Genome sequencing and bioinformatics

Bacteriophage genomic DNA was isolated by phenol:chloroform extraction [58]. Sequencing was performed using the Illumina MiSeq platform (Illumina Inc., USA) at the Nucleomics Core (VIB, Belgium). A custom 2*150 bp paired-end DNA library (Nextera XT sample prep) was prepared with an average fragment length of 500 bp. The quality of each library preparation was verified using the Agilent Bioanalyzer. The library prep was sequenced and reads containing adapters, contamination or low-quality bases were removed in the pre-processing step. Quality trimming of the paired-end data set was performed with CLC Bio Genomics Workbench v7.0 (Aarhus, Denmark) using a quality score of 0.02 and a maximum of 2 ambiguous nucleotides per read. Next, *de novo* assembly was performed with the trimmed paired-end dataset with word and bubble size set at 20 and 50, respectively, a minimum contig length of 200 bp and auto detection of paired distances with scaffolding. This resulted in a single contig with an average coverage of 3,435 x. The assembly was circular with a relatively homogenous coverage.

Bioinformatics analysis was performed on the Biolinux 8 operating system [63]. Open reading frames (ORFs) were predicted using a combination of GeneMark [64], Glimmer 3.02 [65] and Prodigal [66]. Annotation was performed using Artemis [67] and graphical maps were prepared using the CGView Comparison Tool [29]. Nucleotide sequences were queried against the non-redundant database using standard and discontigous megablast. Putative functions for gene products were assigned by querying translated sequences using BLASTP and PSI-BLAST against the non-redundant database [68,69] and by searches against the conserved domain database [70], Pfam [71] and InterPro [72]. Additional functional inferences were obtained using the HH-suite toolset [73,74]. The ExPASy tool Compute pI/Mw [75] was used to predict molecular weight and isoelectric point. Prediction of trans-membrane helices was performed using TMHMM 2.0 [76] and searches for signal peptides were carried out using SignalP [77]. tRNAScan-SE [78] and ARAGORN [79] were used to predict of tRNAs. Searches for regulatory elements, candidate promoter sequences and conserved intergenic motifs were performed using MEME on 100 bp sequences upstream of ORFs [80]. Putative rho-independent terminators were predicted using TransTermHP [81] and candidate terminators were assessed according to location, the presence of a U-rich tail and stable predicted stem loop secondary structure (ΔG ≤ -10 kcal mol^-1^) as calculated by MFold [82].

## Supporting information

S1 File Restriction digests of *Acinetobacter* bacteriophage Loki DNA. (PDF)

S2 File Features, BLASTP homologues and presence of conserved domains and motifs of predicted proteins encoded by *Acinetobacter* bacteriophage Loki. (XLSX)

S1 Table *Acinetobacter* spp. assessed by spot plate assay and efficiency of plating (EOP). (PDF)
