# Supplementary material for: Characterisation and genome sequence of the lytic Acinetobacter baumannii bacteriophage vB_AbaS_Loki
Source: PLoS One. 2017 Feb 16;12(2):e0172303. doi: 10.1371/journal.pone.0172303 (PMC5313236; doi:10.1371/journal.pone.0172303)
Supplement: S1 File — (PDF) [file pone.0172303.s001.pdf]

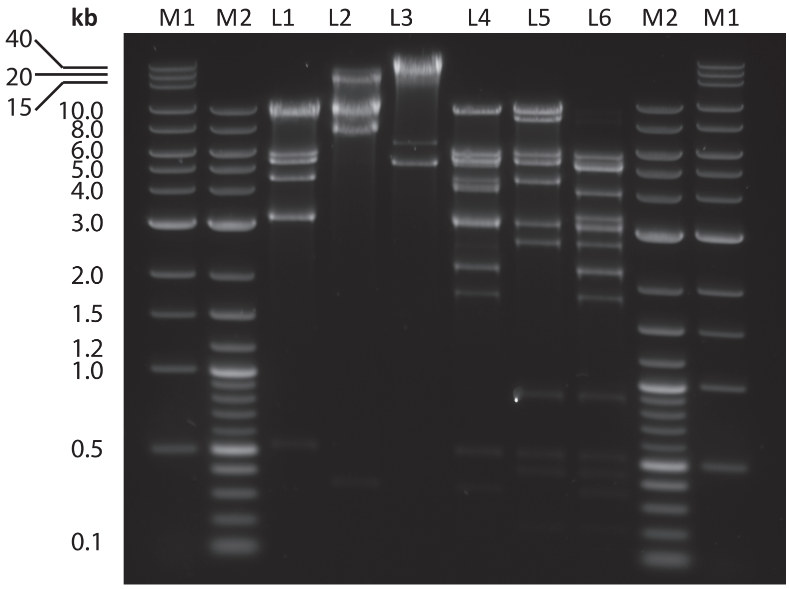

Supplementary Figure A. Restriction digests of Loki genomic DNA. M1, 1 kb extend DNA ladder; M2, 2-log DNA ladder; L1, BclI; L2, BmtI; L3, BsrGI; L4, BclI + BmtI; L5, BclI + BsrGI; L6, BmtI + BclI + BsrGI.

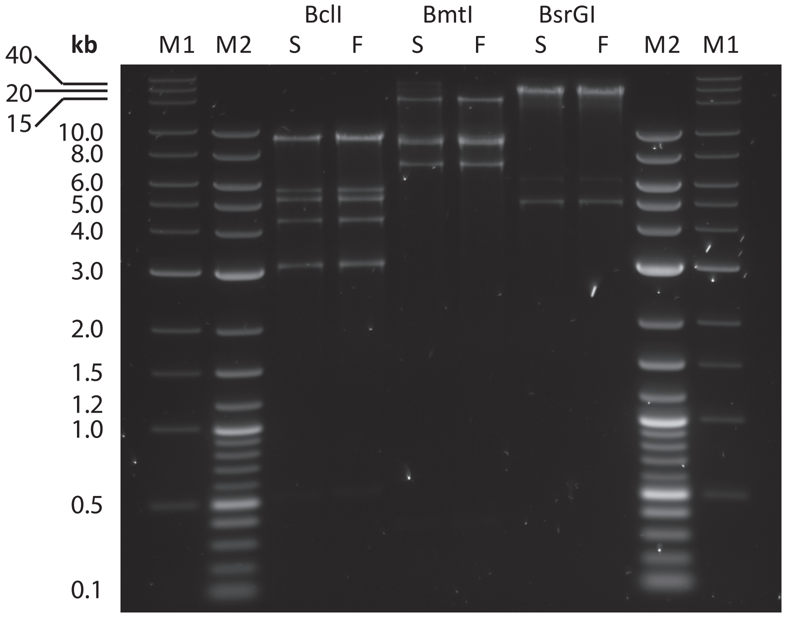

Supplementary Figure B. Cohesive end DNA analysis. Following restriction digestion, DNA was heated to 80°C for 15 minutes, then fast (F) or slow (S) cooled to room temperature. M1, 1 kb extend DNA ladder; M2, 2-log DNA ladder.

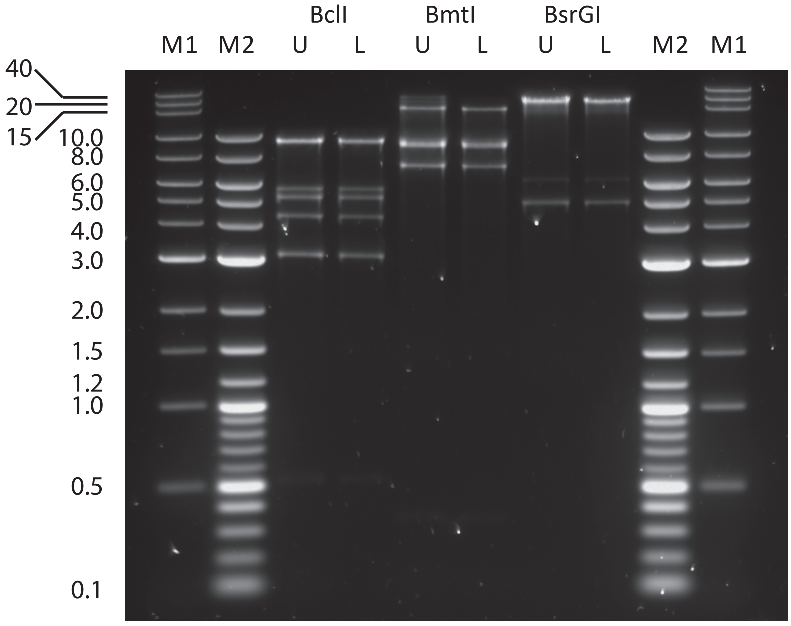

Supplementary Figure C. Restriction digestion of ligated (L) and unligated (U) Loki genomic DNA. M1, 1 kb extend DNA ladder; M2, 2-log DNA ladder.

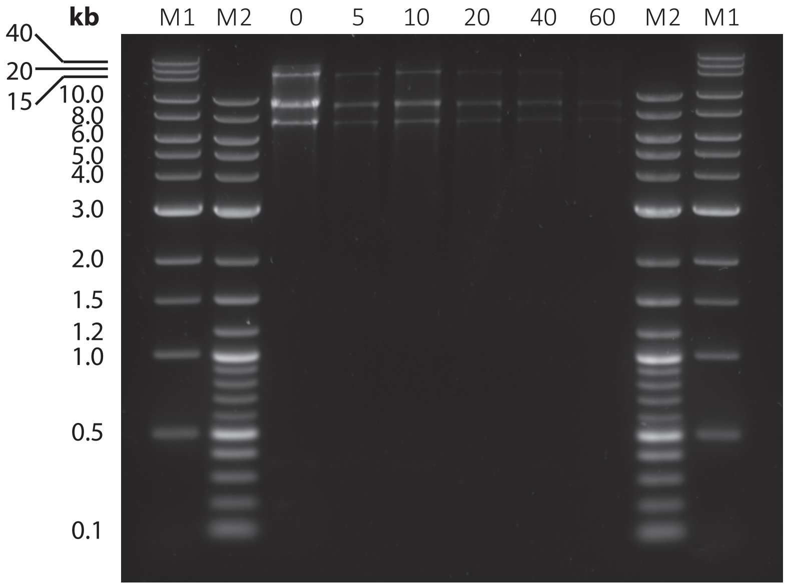

Supplementary Figure D. Time limited digestion of Loki genomic DNA with BAL-31 followed by digestion with BmtI. M1, 1 kb extend DNA ladder; M2, 2-log DNA ladder. Numerals above lanes denote restriction time with BAL31 in minutes.

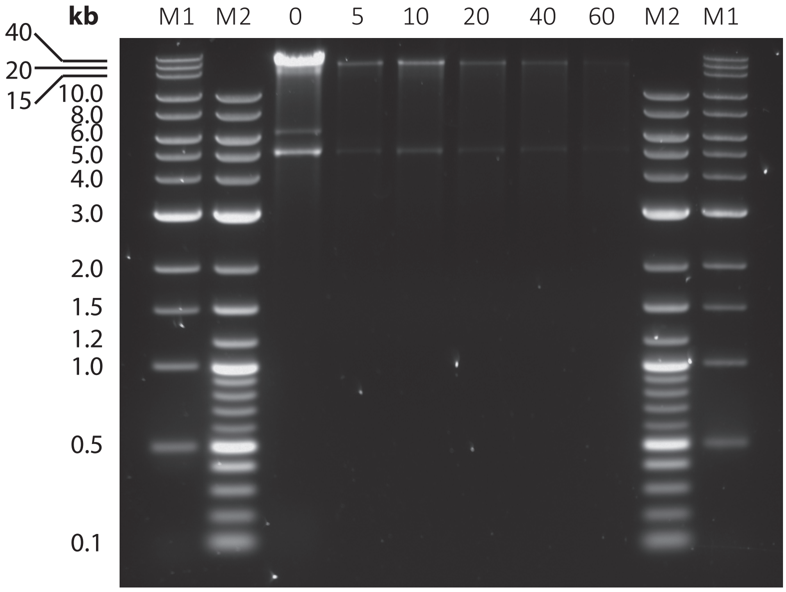

Supplementary Figure E. Time limited digestion of Loki genomic DNA with BAL-31 followed by digestion with BsrGI. M1, 1 kb extend DNA ladder; M2, 2-log DNA ladder. Numerals above lanes denote restriction time with BAL31 in minutes.

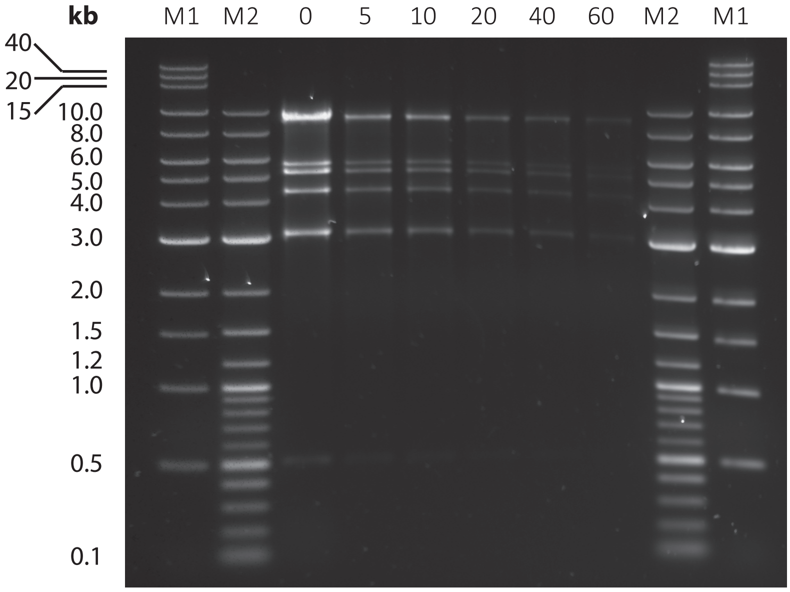

Supplementary Figure F. Time limited digestion of Loki genomic DNA with BAL-31 followed by digestion with BclI. M1, 1 kb extend DNA ladder; M2, 2-log DNA ladder. Numerals above lanes denote restriction time with BAL31 in minutes.

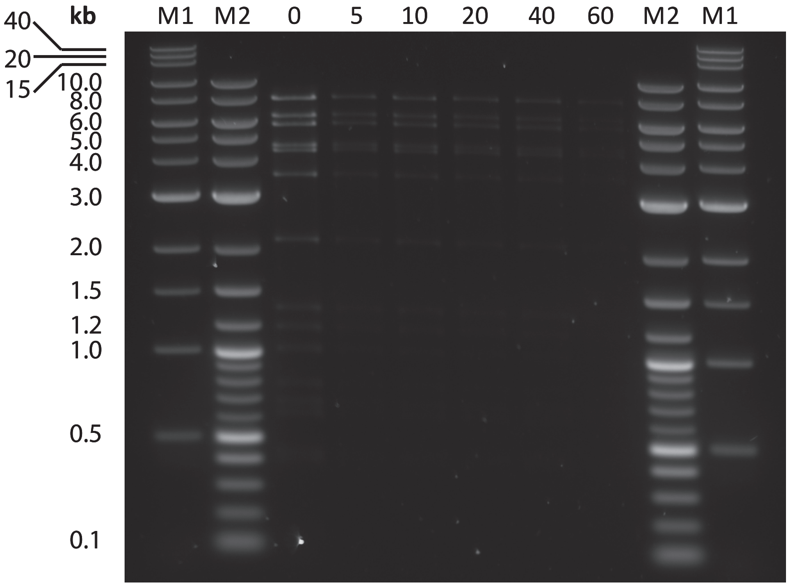

Supplementary Figure G. Time limited digestion of Loki genomic DNA with BAL-31 followed by digestion with SspI. M1, 1 kb extend DNA ladder; M2, 2-log DNA ladder. Numerals above lanes denote restriction time with BAL31 in minutes.
