# Supplementary material for: Characterisation and genome sequence of the lytic Acinetobacter baumannii bacteriophage vB_AbaS_Loki
Source: PLoS One. 2017 Feb 16;12(2):e0172303. doi: 10.1371/journal.pone.0172303 (PMC5313236; doi:10.1371/journal.pone.0172303)
Supplement: S1 Table — (PDF) [file pone.0172303.s003.pdf]

Supplementary Table 1. *Acinetobacter* sp. assessed by spot plate assay and efficiency of plating (EOP). Spot; + zone of lysis present, - no lysis observed. EOP; ratio of titre on test strain to titre obtained on propagating host as determined by overlay plaque assay, - no plaques observed. CL: International clonal lineage; CC: clonal complex; NA: not attributable.

***Acinetobacter* isolate**	**Relevant characteristics**	**Source**	**Spot assay**	**EOP**
A. baumannii AYE	Dark colony morphotype; CL-I	ATCC BAA-1710	+	-
A. baumannii AYE	Light colony morphotype; CL-I	ATCC BAA-1710	+	-
A. baumannii SDF	-	ATCC BAA-1709	-	-
A. baumannii CL 330/86	Sporadic; NA	NCTC 12156	+	-
A. baumannii	Sporadic; NA	ATCC 17978	+	1
A. baumannii	Colistin-resistant; Sporadic; NA	ATCC 17978 *lpxA*	-	-
A. baumannii-calcoaceticus SM37212	-	North Bristol NHS Trust	+	-
A. baumannii	OXA-23 clone 2	NCTC 13421	+	-
A. baumannii	OXA-25; CL-II	NCTC 13302	+	-
A. baumannii T-strain	OXA-51-like; CL-II	NCTC 13423	+	-
A. baumannii 3516/60	NA	NCTC 10303	+	-
A. baumannii	OXA 23 clone 1	NCTC 13424	+	-
A. baumannii W-strain	CL-1	Public Health England	+	-
A. baumannii UKA1	SEM clone; C-LII	Public Health England	+	-
A. baumannii UKA2	BAC-1; CL-II	Public Health England	+	-
A. baumannii UKA3	SEM clone; CL-II	Public Health England	+	-
A. baumannii UKA4	-	Public Health England	*	-
A. baumannii UKA5	EAC-6; CL-II	Public Health England	+	-
A. baumannii UKA6	FAC-25; NA	Public Health England	-	-
A. baumannii UKA7	OXA-23 clone 1	Public Health England	+	-
A. baumannii UKA8	SEM clone; CL-II	Public Health England	+	-
A. baumannii UKA9	Unique; NA	Public Health England	+	-
A. baumannii UKA10	OXA-23 clone 1; CL-II	Public Health England	+	-
A. baumannii UKA11	GAC-9; NA	Public Health England	+	-

A. baumannii UKA12	FAC-24; CL-I	Public Health England	+	-
A. baumannii UKA13	FAC-24; CL-I	Public Health England	+	-
A. baumannii UKA14	GAC-9; NA	Public Health England	+	-
A. baumannii UKA15	GAC-9; NA	Public Health England	+	-
A. baumannii UKA16	FAC-16; CL-I	Public Health England	+	-
A. baumannii UKA17	Midlands 2; CL-III	Public Health England	+	-
A. baumannii UKA18	AAC-1; CL-I	Public Health England	+	-
A. baumannii UKA19	NW strain; CL-II	Public Health England	+	-
A. baumannii UKA20	-	Public Health England	+	-
A. baumannii A318	CC113	Universidad de Buenos Aires	+	-
A. baumannii A118	Naturally competent	Universidad de Buenos Aires	+	-
A. baumannii 211	-	Public Health England	+	-
A. baumannii A600	Colistin resistant; CL-I/CC109	Universidad de Buenos Aires	+	-
A. baumannii A601	Colistin sensitive; CL-II	Universidad de Buenos Aires	+	-
A. baylyi ADP-1	-	ATCC 33305	+	-
A. lwoffii	-	NCTC 7976	+	-
A. calcoaceticus	-	NCTC 7461	+	-
A. baumannii-calcoaceticus 39558	CL-I/CC109	Universidad de Buenos Aires	-	-
